# The Impact of Job Stress, Role Ambiguity and Work–Life Imbalance on Turnover Intention during COVID-19: A Case Study of Frontline Health Workers in Saudi Arabia

**DOI:** 10.3390/ijerph192013132

**Published:** 2022-10-12

**Authors:** Mohammed Alblihed, Haitham Ali Alzghaibi

**Affiliations:** 1 Department of Medical Microbiology, College of Medicine, Taif University, P.O. Box 11099, Taif 21944, Saudi Arabia; 2 Department of Health Informatics, College of Public Health and Health Informatics, Qassim University, Al Bukayriyah 52741, Saudi Arabia

**Keywords:** burnout, work–life imbalance, turnover intention, COVID-19, KSA

## Abstract

The main objective of the present study is to examine the impact of job stress, role ambiguity, work–life imbalance and burnout on employee turnover intention. Moreover, the mediating role of burnout between job stress, role ambiguity, work–life imbalance and turnover intention is also examined. The data collection for this quantitative research was conducted through the “Questionnaire” technique. The questionnaire was developed based on previously established questions available in the literature. The data were collected using simple random sampling from the healthcare workers of KSA. From the distributed questionnaire, 73.5% of the usable questionnaires were returned. This study used SPSS and PLS for the analysis of the data to highlight the most significant variables that impact the employees’ turnover intentions among KSA health workers. The findings show that job burnout is clearly related to turnover intentions and is positively affected by both role stress and role ambiguity. Moreover, a statistically positive association is found between work–life imbalance and burnout among the healthcare workers in KSA. Furthermore, the mediating role of burnout is also confirmed in this study. The study also indicates that role ambiguity and role stress due to COVID-19 may create burnout among employees, which may lead to turnover intention among healthcare workers. There is a lack of research on the assessment of the impact of the novel COVID-19-related job stress, role ambiguity and work–life imbalance on the medical staff’s turnover intentions in hospitals. This study fills the gap of the limited studies conducted regarding the identification of the factors that can create turnover intention among healthcare workers of KSA by providing empirical evidence from a Gulf country, Saudi Arabia. This study provides managerial implications for hospital management and health policymakers to develop a strategy to retain the employees. Furthermore, healthcare administrators need to pay close attention to front line workers’ turnover intentions as these medical heroes are the vital part of our society who assist patients to receive their initial treatment during the COVID-19 pandemic.

## 1. Introduction

Employee turnover is a phenomenon that has negative implications and is potentially costly for organizations. On the other hand, there is another type of turnover, which includes termination, involuntarily and resignation. The impact of turnover on an organization is negative because of disruption in communication and social structure, the loss of an employee who may be highly skilled or performing very well, the loss of production during the search for the replacement of employee, searching for new employees as well as the provision of training and high recruitment cost. On the other hand, the turnover of employees not only damages the organization in terms of advertising cost, selection cost, recruitment cost and termination cost, but the turnover of any employee can impact the other employee’s ability to perform their designated tasks [1,2].

Employees working in any organization may face mental as well as bodily consequences because of job-related psychological pressure as well as work pressure and job burnout. Job burnout is a term that has been discussed by a number of researchers in recent years. The basis of job burnout is the constant pressure asserted by the employer on the employee. Due to this constant pressure, employees feel that they have less energy. They also feel the need to do something to preserve their position. It has been reported by a number of researchers that employees facing job burnout have the tendency to underperform in the job, and they also develop the intention to leave their workplace. Researchers have been interested in job burnout’s effects because of two main reasons. Firstly, leaders within our workplace are responsible for the well-being of the employees. Secondly, the well-being of employees has an impact on the performance of an employee, which, in turn, eventually has an impact on the overall performance of the entire organization [3].

A number of employees feel job stress because of a number of job-related reasons, also known as environmental stimuli. At present, job stress among employees creates a low morale among workers and is more frequent as well. Employees working in an organization may face job stress because of a number of employer-related factors, such as competitive environment, technological changes, low salary, lack of recognition, lack of motivation, overwork, workload and social factors as well. The fact that employees feel stressed can have advantages as well as disadvantages at the same time. Sometimes, job stress may prove to be productive for the employees as it may have a positive impact on employee performance. On the other hand, it also has the potential to damage the employee performance. It has the tendency to be productive for the employee as the employee may become motivated to perform well. As a result, the productivity of the employee will be enhanced. On the other hand, it can be counterproductive for the employee if the employee feels pressure, and he/she may not produce something concrete and of good quality. At present, job stress is increasing in the lives of employees, which damages their performance [4]. Currently, employees are also feeling stressed because of the COVID-19 pandemic. Employers are focusing on the adoption of steps through which they can reduce the cost of business. One of these steps is reducing the salaries of employees, which creates stress among employees and leads to job burnout.

Employees also face role ambiguity, which is mainly caused because of a dysfunctional organizational structure. Employees facing the issue of role ambiguity often faces difficulties in finishing the assigned task. Role ambiguity often creates job burnout because of dissatisfaction with the job. It is because the competence of the individual is challenged to finish the job. The situation in which an employee cannot finish the assigned task often leads to anxiety in the workplace. Thus, because of role ambiguity, a person is unable to finish the assigned task. One of the negative outcomes of role ambiguity is the decrease in job satisfaction of the employee [5].

Employees working in an organization often face the issue of work–life balance as well. This concept has gained the attention of a number of researchers in recent years. Work–life balance is referred to as the desired and actual proportion of the activities of an employee in his/her private and work life. In the definition of work–life balance, the term actually is referred to as the total proportion of time allocated to activities of work and the activities of one’s personal life. On the other hand, researchers have placed importance on work–life imbalance because it creates considerable problems for the employees in terms of declining levels of productivity, workplace monotony and the health of the employee. Because of imbalance, there are often consequences on the personal life of the individual, enhancing the percentage of divorces and causing other family issues [6]. Therefore, this paper examines the role of job ambiguity, stress caused by COVID-19 and work–life imbalance on job burnout. Moreover, the mediating role of job burnout is also examined between work–life imbalance, job ambiguity, stress and turnover intention.

A self-administered survey using the “Questionnaire” technique was conducted to assess the turnover intentions of health workers who were exposed to the threat of COVID-19, in terms of burnout, role stress, role ambiguity and work–life imbalance leading to employee turnover intention.

This paper extends the literature by exploring the relationship of burnout and job stress, role ambiguity and work–life imbalance with turnover intentions during the COVID-19 pandemic. The results of the study may contribute to having a better understanding of the phenomena of emotional exhaustion and job stress during COVID-19. This paper also intends to understand the mechanisms behind the unfavorable conditions that contribute to an employee’s turnover intentions. Additionally, the importance of these findings in extending social exchange theory concepts to the field of employee turnover intentions is examined.

This study provides a clear understanding of the inter-relationships between the various factors affecting turnover intention, which can be used by the emergency departments and medical organizations to develop and improve policies and practices aimed at increasing job retention among health workers. Managerial committees at hospitals need to pay attention to the staff’s job stress, role ambiguity and burnout in association with their involvements in the nationwide COVID-19 breakout [7].

Although there are few studies available regarding nurses’ turnover intention focusing on this phenomenon in normal situations [8,9,10], there are no papers available regarding frontline workers’ turnover intentions due to burnout related to job stress, work–life imbalance and role ambiguity experienced during COVID-19. Thus, this study contributes to the body of knowledge by assimilating scattered academic work into one framework.

## 2. Literature Review

The novel Coronavirus 2019, also known as COVID-19, has caused considerable panic around the world and has affected the general public by causing damage to public health and losses in the financial and economic sectors worldwide [11]. Consequently, the World Health Organization confirmed it a global health emergency on 30 January 2020 [12]. The nature of the disaster caused thousands of frontline and health workers to be positioned in infected regions in order to rescue, manage and control the infection of COVID-19. Job stress is considered as one of a consequence of COVID-19 [13,14,15] that has an impact on the medical profession.

The term job stress has been defined by a number of researchers in the previous literature. It is defined as a set of external factors that are harmful in the workplace. These factors can be social, physical and psychological. Job stress is the reaction of the individual to the workplace environment, which can be physically, emotionally and morally threatening. Employees face stress at their workplace when there is an imbalance between the employee’s working ability and the demand for work. Stress can also be an indicator of overload, conflict and ambiguity arise from the work environment, including individual characteristics. There are three main components of stress. The first one is the stimulus, which means the primary stimulant that results from stress feelings, which can be caused by the environment, individual or organization. The second one is known as a response, which is the behavioral, physical and psychological reaction that is represented by the individual in the form of frustration and anxiety [16]. In the end, the third one is the reaction, which explains the relationship between stimulus and response relationship [17].

Employees, especially those working in the medical sector, face the issue of stress because of medical issues as the life of employees working in medical institutes is in danger as well. Moreover, these employees are also under great pressure to save the lives of other patients. These employees are exposed to an environment that has low resources and a high demand for the job. Therefore, there is a high level of stress. Because of this high level of stress, the lives and health of the employees are considerably impacted as well [14,18].

### 2.1. Role Ambiguity

Individuals face the issue of ambiguity in a role when the roles are not clear, or they are poorly defined by the organization. Because of role anticipation, the performance of the individual deviates. The main reason is that the expectations of the role are not clear. For instance, the list of tasks that are performed by the individual corresponds to a specific job description. This job description mostly includes the responsibilities to be performed by the individual, functions and general tasks to be completed by that certain employee. Sometimes, the job description also includes the specification of the job and to whom that individual needs to report regarding daily activities. The problem of role ambiguity arises when an individual’s job is not clearly defined, when the expectations, procedures and requirements are not fully clear to the employee [19].

For this reason, role ambiguity is defined by researchers as a lack of satisfactory information, which is important for someone to fulfill the requirements of the job in a satisfactory manner. Therefore, an employee faces the issue of role ambiguity when he/she does not have the required information regarding their duties. Important information required for the job includes (i) relevant expectations from the employee, (ii) important activities to be performed to fulfill the job requirements, (iii) the result of fulfilling or not fulfilling the job requirements, (iv) which behavior is punished or compensated and (v) advancement opportunities [20].

### 2.2. Work–Life Imbalance

In a broader sense, work–life balance covers all the aspects of the work life and personal life of the employee. This shows that the work–life balance should be placed on society, community, workplace, family and individual as a whole. Past studies have defined work–life balance as “the amount of time and the degree of satisfaction with the work and family role”. On the other hand, employees usually face work–life imbalance when the boundaries between their personal and professional life are very thin, or they are not very clearly described by the employer. Researchers have explained that it is key to keep the focus on the domain of family and work because work and family are both very important parts of anyone’s life [21]. Any demand that competes in terms of work will have a negative impact on the well-being of the employee. Researchers in past studies have agreed that the work–life balance is key to keep employees satisfied and maintain harmony in the workplace. On the other hand, work–life imbalance causes high absenteeism, a decrease in productivity and increase depression among the employees. Another consequence of a work–life imbalance is the impact on the morale of employees [22]. As a result, organizations experience poor quality of work, low productivity and high turnover. In the same vein, Malik M.I. et al. [23] pointed out that work–life imbalance is also the cause of high levels of stress among employees.

### 2.3. Job Burnout

The term burnout is mainly explained as the condition that is the outcome of work overload. It mostly occurs when an individual does not serve the purpose of the job. Researchers have defined the term burnout as the psychological state of the individual, which is caused by exhaustion. It includes, most of the time, a combination of negative attitude, low motivation level, ineffectiveness at work and stress. Researchers have also defined it as the psychological state that is mostly caused by job-related stress factors. In the previous literature, different types of state of job burnout have been identified, e.g., emotional exhaustion and depression [24].

The emotional demand of the job of the individual creates emotional exhaustion. At the same time, depression is referred to as the individual’s negative behavior that needs care and is serviced. In fact, few authors have treated exhaustion as a dimension of burnout, which is the outcome of a number of stress-related factors. These factors may include psychological and physical ones [25]. Presently, one of the psychological factors that employees face is COVID-19. Employees working in a number of organizations are feeling exhausted because of the psychological pressure caused by COVID 19. Thus, burnout has had an impact on a number of occupations.

### 2.4. Turnover Intention

Turnover intention is the likelihood of an employee to switch or leave their current job with the current employer. Every type of organization is at great risk regarding the turnover intention of the employee, regardless of the size of the organization or its location. Employee turnover intention shows the plan of the employee to leave the job voluntarily. Voluntarily turnover is the plan of the employee to leave the job. There is a close relationship between employee turnover intention and actual turnover because behavioral turnover is mainly dependent on turnover intention [26]. Therefore, turnover intention is essentially the determinant of turnover behavior. It is critical to understand the turnover intention of the employees in order to identify the reasons that may cause the employees to leave a job [27].

### 2.5. Burnout and Turnover Intention

In the past, a number of researchers have conducted research regarding the impact of job burnout on turnover intention. In this regard, Lin Q.H. et al. [28] mentioned that burnout among employees creates a number of withdrawals, including an increase in absenteeism and willingness to switch jobs. In the same context, Goodman [29] pointed out that employees who have a high intention of switching jobs have a high level of job burnout as well. On the basis of the above discussion, we hypothesize that:

**H1.**  *Job burnout has a positively impact on employee turnover intention*.

### 2.6. Role Stress and Burnout

A number of past studies show that there exists a positive impact of job stress on the burnout of the employee. In this context, researchers have reported that the work overload among employees causes burnout. The factors that create job stress among employees have a positive relationship in the creation of job burnout among employees. Employees who have work overload or stress due to any other psychological reasons such as COVID-19 can become exhausted. As employees have high levels of exhaustion, it will result in a high level of intentions to leave the job [30]. Past studies have provided evidence of the role of stress on turnover intention and burnout, as employees who experience work overload are most likely to feel exhausted emotionally. On the other hand, research conducted by Zhan Y. et al. [31] also revealed the impact of job stress on burnout.

**H2.**  *Role stress is positively related to burnout.*

**H3.**  *Burnout is a significant mediator between role stress and turnover intention.*

### 2.7. Role Ambiguity and Burnout

Employees who lack information regarding their job can experience role ambiguity. In this situation, employees are not sure what roles they need to perform to fulfill the requirements of the job. Thus, a number of problems can be faced due to role ambiguity, such as decreased involvement in the job, depression and anxiety. Psychological strain, also known as burnout, is one of the outcomes of role ambiguity. Employees who are dissatisfied with the job often face burnout. Overall dissatisfaction among employees may cause the feeling to finding ways to deal with stress or change. Researchers have pointed out that job burnout is the outcome of role stress. In the workplace, researchers have pointed out role ambiguity as an important reason to cause stress among the employees. Basically, role ambiguity is the level of disinformation that is experienced by the employee or the lack of information related to the task assigned to the employee. A number of antecedents in terms of personal variables and organizational variables are reported as consequences and antecedents of job burnout. Among these, role ambiguity is pointed out as a critical predictor [32].

**H4.**  *Role ambiguity is positively related to burnout.*

**H5.**  *Burnout is a significant mediator between role ambiguity and turnover intention.*

### 2.8. Work–Life Imbalance and Burnout

Researchers have pointed out that there is a positive association between burnout and work–life balance. If the job resources and job demand are increased to one point, the job burnout condition will be impacted by around 25%. The disproportion among work life and work exists, and it has a significant impact on burnout, depersonalization, and exhaustion [33]. Therefore, among the other antecedents of burnout, work–life imbalance recorded to be the most influence factor t. Moreover, researchers also pointed out that work–life imbalance may cause stress in the life of the employee related to the job [34].

**H6.**  *Work–life Imbalance is positively related to burnout.*

**H7.**  *Burnout is a significant mediator between work–life imbalance and turnover intention.*

## 3. Methodology

A cross-sectional design was used to conduct this study in order to assess the turnover intentions of health workers who were exposed to the threat of COVID-19-related role stress, role ambiguity and work–life imbalance. This study used a quantitative approach because past quantitative studies have concluded that nurses and other medical staff who dealt with coronavirus-infected patients were more likely to experience psychological issues, such as stress, restlessness, anxiety and depression [35,36]. The same is applicable to this study as it aims to explore the impact of burnout caused by COVID-19 on medical staff turnover intentions in Saudi Arabia.

Data were collected from healthcare workers who worked in the public and private sector hospitals across the Kingdom of Saudi Arabia, mainly from major cities including Medina, Jeddah, Riyadh, Abha and Taif, from March 2022 to April 2022, by using simple random sampling because it can reduce biase in sampling and assist to select sample that represent the population [37,38]. Members from medical staff were selected from the ICUs, isolation wards, emergency departments, respiratory wards and infection control offices. An informed consent was provided to the participants and we ensured their privacy and confidentiality.

## 4. Instrument and Measurement

A self-administered survey was conducted to collect data using the “Questionnaire” technique. Questionnaires are considered to be a vital tool of data collection within work-related, health and well-being literature [39], and are suitable for measuring individuals’ attitudes. In this study, the questionnaire was divided into three parts. The first contained the demographic information of the employees. The second part consisted of the employees’ perception and problems related to job stress, role ambiguity and work–life imbalance caused by the COVID-19 pandemic; and the third part relates to organization outlook and policies related to employees. The questionnaires were mostly distributed by personally visiting the hospitals, except for a few that were sent via email or by post. A 5-point Likert scale, ranging from 1 = strongly disagree and 5 = strongly agree, was used to assess the responses.

### Measurements

Role ambiguity amongst health workers was measured using the Bowling Scale for Role Conflict and Ambiguity. This scale is composed of the following 6 statements: “I am not sure what is expected of me at work”, “The requirements of my job aren’t always clear”, “I often don’t know what is expected of me at work”, “I know everything that I am expected to do at work with certainty, “My job duties are clearly defined”, and “I know what I am required to do for every aspect of my job.” Burnout was tested using the emotional subscale of the Maslach Burnout Inventory General Survey [40]. The scale contains five items, which measure being emotionally over giving and exhausted by one’s work. A sample item is “I felt tired when I got up in the morning and had to face another day on the job”. Health workers’ turnover intentions were evaluated using the adapted scale from Farh et al. [41]. The scale consists of 4 items. A sample item is “I often think of quitting my present job”.

Initially, 522 questionnaires were distributed among the respondents. A total of 397 were usable, with a response rate of 75.86%. Unanswered questionnaires were excluded, and normality was established. The received questionnaire was punched in SPSS for initial analyses, such as multi-collinearity screening, detection of outliers, missing value analysis and descriptive analysis. For further analysis, PLS 3.3.2 was used by the researchers.

The consistency of the answers within each of the scales was examined; this is often referred to as the internal reliability of the scales. It is essential that such scales are highly reliable to ensure that all items within each of the scales measure the same thing. Reliability, in this paper, was measured through Cronbach’s alpha test, which measures consistency in terms of percentages ranging from 0% to 100% (0–1). Reliability scores above 70% were considered acceptable.

## 5. Results and Analysis

The data gathered from the respondents were entered into SPSS for preliminary analysis. Initial tests, such as multi-collinearity, detection of outliers, normality tests and descriptive statistics, were conducted. Subsequently, we conducted a demographic analysis using SPSS. According to the findings, more than 73% of the healthcare workers were female. Most of the respondents were married, constituting around 84%. More than 35% of the respondents were aged 40 years and over, whereas 33% were aged 20–30 years. Most of the respondents, around 51.2%, were master’s degree holders (see Table 1).

**Figure 1:**
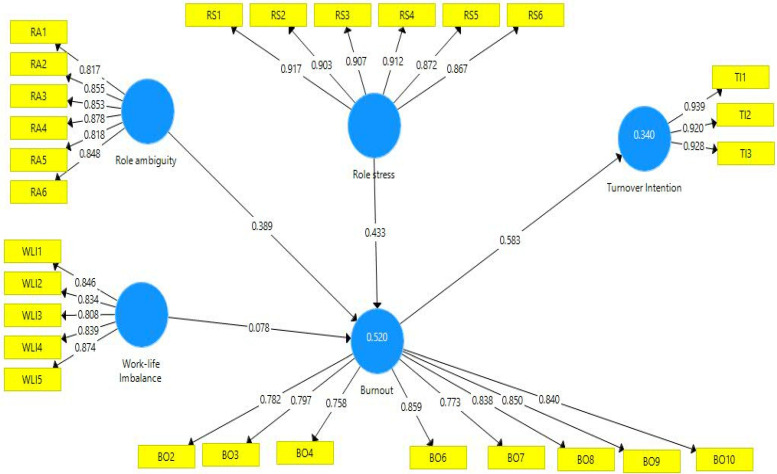
Measurement model used in the present study. Note: BO = Burnout, WLI = Work–life imbalance, TI = Turnover intention, RS = Role stress, RA = Role ambiguity.

Based on the recommendations of Henseler J. et al. [42], a two-step approach was followed in the present study to run the PLS-SEM. The first step was to assess the measurement model, which led to the examination of the structural model. The following shows the measurement model used in the present study (see Figure 1).

The main purpose of evaluating the measurement model was to assess the reliability and validity of the data. The first phase was to assess the reliability of inter items, which is conducted through the evaluation of factor loadings. In this respect, Hair J.F. et al. [43] stated that the threshold value should be a minimum of 0.70 [44]. Table 2 shows the reliability of the items of all variables used in the present study fulfilled the criteria.

In the next step after the successful evaluation of the reliability of the items, the examination of the convergent validity was conducted. For this purpose, we evaluated AVE by ensuring that all values of AVE were above the threshold level of 0.50 [44,45]. Additionally, this research also assessed the values of Cronbach’s alpha and the composite reliability, for which the minimum acceptable value must be greater than 0.70 [46]. The results shown in Table 3 reflect the minimum threshold level that was fulfilled in the present study.

The discriminant validity of the data is confirmed when the loading of the items is high on its own construct. Therefore, in the present study, the discriminant validity was assessed through the differential of square of values of AVE of every construct through all other constructs, which has to be higher than the correlation among two factors [47]. Therefore, Table 3 shows that the correlation values among each construct with itself is more than all remaining constructs, showing that the discriminant validity of all constructs is fulfilled.

The heterotrait–monotrait ratio was used in the present study to assess the discriminant validity of the present study (see Table 4). The multritrait multimethod is the method that is the base of HTMT [48]. In this context, Ramayah T. et al. [49] showed the minimum threshold value for the discriminant validity if the value is less than 0.85 (following strict criteria) and 0.90 (following lenient criteria). Table 5 shows the strict criteria of HTMT is fulfilled in the present study.

After the assessment of the measurement model, we evaluated the structural model in order to confirm the relationship among the proposed relationships. In this step, the guideline of Henseler J. et al. [42] was followed to examine the significance of the coefficient. A total of 5000 subsamples were used in the present study to perform bootstrapping for structural model [43]. The results in Table 6 show the findings of the direct hypothesis.

The hypotheses were tested through two steps using structural equation modeling (in AMOS). Firstly, H1, H2, H4 and H6 were tested, and the model was subjected to multiple satisfactory goodness of fit indices values (enter value).

The results in Table 6 of the proposed relationship are supported statistically. According to the results, BO is positively related to TI (Beta = 0.583, t = 14.161); thus. H1 is supported. Additionally, BO is positively affected by RS, supporting H2 (Beta = 0.433, t = 7.758). Furthermore, BO is positively affected by RA (Beta = 0.389, t = 7.212), supporting H4. In the end, the H5 proposed in the present study is also supported because WLI and BO are also statistically positively associated with each other (Beta = 0.078, t = 1.801).

**Figure 2:**
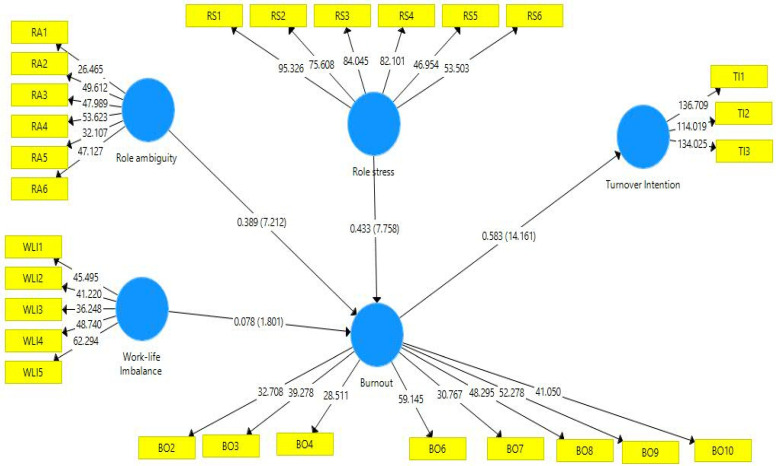
Structural model of the present study. Note: BO = Burnout, WLI = Work–life imbalance, TI = Turn-over intention, RS = Role stress, RA = Role ambiguity.

In the next step, H3, H5 and H7, which proposed indirect effects, were also subjected to multiple satisfactory goodness of fit indices (enter values). Moreover, mediation results are also presented in Table 7. According to the findings, BO mediates the relations of RS and TI (Beta = 0.227, t = 6.038), supporting H3. Moreover, H5, proposed in the present study, is also supported because BO mediates the relationship of RA and TI (Beta = 0.253, t = 6.911). In the end, BO mediated the relationship of WLI and TI supporting H7 (Beta = 0.045, t = 1.819). Figure 2 shows the structural model of the present study.

In this study, R^2^ was used to assess the predictive power of the power. The PLS algorithm was used to compute R squared in the present study. The values of R squared in the present study are 0.52 for BO and 0.34 for TI. According to Falk and Miller [50], the value of R squared is acceptable if it is greater than 0.10. This criterion is fulfilled in the present study. Moreover, predictive relevance was conducted in the present study through the assessment of Q squared. Blindfolding procedure was adopted in the present study for the assessment of Q squared. Predictive relevance is established if the value of Q squared is greater than zero. The value of Q squared in Table 8 shows the criteria are fulfilled in the present study [51].

## 6. Discussion

Employees are an asset to any organization. In the times of COVID-19, it is very important for the schools to develop a strategy by which they can retain employees. For this reason, it is vital to identify factors that may create burnout among employees due to COVID-19, and employees may tend to leave the job. In order to achieve this aim, the study was conducted to examine the role of role ambiguity, work–life imbalance and role stress on the turnover intention of healthcare workers through job burnout due to the COVID-19 pandemic. The findings show that job burnout is positively related to work–life imbalance. Or, in other words, it would be correct to say that, if an employee is not able to keep a balance between their personal and professional lives, the employee will face job burnout, which will lead to turnover intention.

The findings also suggest that COVID-19-related role stress has a significant effect on medical workers’ turnover intentions. Moreover, COVID-19-related job and role stress induced nurses’ turnover intentions. Additionally, the constant arrival of patients after the outbreak of such an infectious disease, that is, COVID-19, caused burnout and stress, which in turn increased turnover intentions among healthcare workers. These findings are consistent with the findings of the previous research of Zhang et al. [52] and Said and El-Shafei [53], who found that job stress has a direct connection with turnover intentions among nurses. It has also been observed by Haas et al. [54] that, during different kinds of viral spreads, nurses become the victims of job stress and they prefer to leave their jobs.

This study’s other finding was that role ambiguity may create burnout among employees, which may lead to turnover intention among healthcare workers. Our results show consistency with a previous study conducted among 170 medical doctors and 81 nurses from one university hospital in Turkey, which revealed a strong positive relationship between role conflict and role ambiguity and work stress, and role conflict and ambiguity could explain higher levels of work stress reported by nurses than by doctors [55].

Although there have been a number of research studies investigating the effect of burnout on turnover intentions, an important contribution of this study is that it investigated the relationships with role ambiguity, work–life imbalance and role stress during the COVID-19 outbreak in the context of Saudi Arabia. The primary contribution of this study is that it provides empirical evidence from a Gulf country, Saudi Arabia, from hospitals working under the threat of COVID-19 and targeting frontline workers who are dealing with COVID-19-related burnout.

### 6.1. Implications

The results of this study have some important implications for health leaders and hospital management, especially to deal with emergency situations such as the COVID-19 pandemic. During such emergencies, healthcare units must prioritize the need to understand the mental health response of health workers. Hospital policies must be reviewed to identify possible sources of role conflict and ambiguity to avoid work stress among frontline workers during emergency situations. Medical administrators must review their selection criteria by focusing on factors such as psychological state, emotional stability and the ability of coping with stress of nurses hired during the time of crises. Further, it is suggested that more medical equipment and facilities be provided to nurses and other healthcare workers so that they feel more appreciated by the institutes, which will eventually reduce their intentions to resign. More social and psychological support should be provided to healthcare workers by the hospitals. The continuous assessment of role ambiguity needs to be executed to identify any problems or difficulties that might affect the stress levels of nurses in hospitals, which consequently affects the quality of the healthcare services provided and employee turnover intentions. In addition, coping management strategies should be introduced by hospital management to decrease turnover intentions related to pandemic outbursts. This research indicates to healthcare managers that a hospital’s long-term strategy should integrate educational programs for management to provide adequate support to medical workers in order to deal with pandemic-like situations with more resilience.

### 6.2. Limitations and Future Recommendations

This study has a variety of limitations, which require future research. Firstly, this study was cross-sectional in nature; in the future, a longitudinal design or experimental studies can be conducted. Secondly, this study was carried out in a country in the Gulf region, so future studies can be carried out in other developing or developed countries to further validate the proposed relationships under the special circumstances of the COVID-19 pandemic. Further studies are needed to illuminate other possible effects of role conflict and ambiguity on the quality of medical care and the outcomes of COVID-19 management. Researchers in future studies may extend the model to the behavior of the employee, such as employee loyalty. Moreover, the moderation of employee trust may also be tested using the present model.

## 7. Conclusions

The findings of this study show that job burnout is clearly related to turnover intentions and is positively affected by both role stress and role ambiguity. Moreover, a statistically positive association is found between work–life imbalance and burnout among the healthcare workers in KSA. Furthermore, the mediating role of burnout is also confirmed in this study. The study also indicates that role ambiguity and role stress due to COVID-19 may create burnout among employees, which may lead to turnover intention among healthcare workers. There is a lack of research on the assessment of the impact of the novel COVID-19-related job stress, role ambiguity and work–life imbalance on the medical staff’s turnover intentions in hospitals. This study fills the gap of the limited studies conducted regarding the identification of the factors that can create turnover intention among healthcare workers of KSA by providing empirical evidence from a Gulf country, Saudi Arabia. This study provides managerial implications for hospital management and health policymakers to develop a strategy to retain the employees. Furthermore, healthcare administrators need to pay close attention to front line workers’ turnover intentions as these medical heroes are the vital part of our society who assist patients to receive their initial treatment during the COVID-19 pandemic.

## Figures and Tables

**Table 1:** Demographic characteristics of the respondents.

Demographic Variables	Category	Frequency	Percentage
Gender	Female	290	73.2%
	Male	107	26.8%
Marital Status	Married	332	83.67%
	Single	65	16.33%
Age	Below 20	0	0%
	20–30	133	33.50%
	31–40	124	31.23%
	Above 40	139	35.01%
Highest Education	Diploma	15	1.2%
	Master’s	192	51.2%
	Bachelor’s	190	47.7%

**Table 2:** Item loading.

	BO	RA	RS	TI	WLI
BO10	0.840				
BO2	0.782				
BO3	0.797				
BO4	0.758				
BO6	0.859				
BO7	0.773				
BO8	0.838				
BO9	0.850				
RA1		0.817			
RA2		0.855			
RA3		0.853			
RA4		0.878			
RA5		0.818			
RA6		0.848			
RS1			0.917		
RS2			0.903		
RS3			0.907		
RS4			0.912		
RS5			0.872		
RS6			0.867		
TI1				0.939	
TI2				0.920	
TI3				0.928	
WLI1					0.846
WLI2					0.834
WLI3					0.808
WLI4					0.839
WLI5					0.874

Note: BO = Burnout, WLI = Work–life imbalance, TI = Turnover intention, RS = Role stress, RA = Role ambiguity.

**Table 3:** Reliability and validity.

	Cronbach’s Alpha	CR	AVE
BO	0.926	0.940	0.661
RA	0.920	0.937	0.714
RS	0.951	0.961	0.804
TI	0.921	0.950	0.863
WLI	0.896	0.923	0.706

Note: BO = Burnout, WLI= Work–life imbalance, TI = Turnover intention, RS = Role stress, RA = Role ambiguity.

**Table 4:** Discriminant validity.

	BO	RA	RS	TI	WLI
BO	0.813				
RA	0.587	0.845			
RS	0.612	0.395	0.897		
TI	0.583	0.526	0.418	0.929	
WLI	0.347	0.340	0.316	0.261	0.840

Note: BO = Burnout, WLI = Work–life imbalance, TI = Turnover intention, RS = Role stress, RA = Role ambiguity.

**Table 5:** Discriminant validity (HTMT).

	BO	RA	RS	TI	WLI
BO					
RA	0.627				
RS	0.649	0.418			
TI	0.629	0.570	0.444		
WLI	0.377	0.373	0.339	0.284	

Note: BO = Burnout, WLI = Work–life imbalance, TI = Turnover intention, RS = Role stress, RA = Role ambiguity.

**Table 6:** Direct results.

HYP	Relationship	Beta	SD	t-Value	*p*-Value	Decision
H1	BO -> TI	0.583	0.041	14.161	0.000	Supported
H2	RS -> BO	0.433	0.056	7.758	0.000	Supported
H4	RA -> BO	0.389	0.054	7.212	0.000	Supported
H6	WLI -> BO	0.078	0.043	1.801	0.036	Supported

Note: BO = Burnout, WLI = Work–life imbalance, TI = Turnover intention, RS = Role stress, RA = Role ambiguity.

**Table 7:** Mediation results.

HYP	Relationship	Beta	SD	t Value	*p*-Values	Decision
H3	RA -> BO -> TI	0.227	0.038	6.038	0.000	Supported
H5	RS -> BO -> TI	0.253	0.037	6.911	0.000	Supported
H7	WLI -> BO -> TI	0.045	0.025	1.819	0.035	Supported

Note: BO = Burnout, WLI = Work–life imbalance, TI = Turnover intention, RS = Role stress, RA = Role ambiguity.

**Table 8:** Q squared and R squared.

	R Squared	Q Squared
BO	0.520	0.338
TI	0.340	0.290

Note: BO = Burnout, TI = Turnover intention.

## Data Availability

The datasets generated and/or analysed during the current study are not publicly available due copywrite and ownership. All primary data collected for this research belong to the researchers. In addition, the dataset includes other data which will be used for another manuscript, but are available from the corresponding author on reasonable request.
